# Assessing decisional conflict and challenges in decision-making among perinatal women using or considering using antidepressants during pregnancy—a mixed-methods study

**DOI:** 10.1007/s00737-023-01341-0

**Published:** 2023-07-22

**Authors:** Fatima Tauqeer, Anne Moen, Kirsten Myhr, Claire A. Wilson, Angela Lupattelli

**Affiliations:** 1 Pharmacoepidemiology and Drug Safety Research Group, Department of Pharmacy, Faculty of Mathematics and Natural Sciences, University of Oslo, Oslo, Norway; 2 Faculty of Medicine, Institute for Health and Society, University of Oslo, Oslo, Norway; 3 Department of Community Medicine and Global Health, Institute of Health and Society, University of Oslo, Oslo, Norway; 4 Section of Women’s Mental Health, Institute of Psychiatry, Psychology and Neuroscience, King’s College London, London, UK; 5 South London and Maudsley NHS Foundation Trust, London, UK

**Keywords:** Mental health, Depression, Pregnancy, Antidepressant, Decisional conflict, Postnatal

## Abstract

This study aims to investigate decisional conflict and elucidate challenges in decision-making among perinatal women using or considering using antidepressant (AD) during pregnancy. A sequential, mixed-methods study was employed among pregnant and postnatal women in Norway who had been offered ADs in the last 5 years. Quantitative data were obtained through an electronic questionnaire. Decisional conflict in pregnancy was assessed using the Decisional Conflict Scale (DCS) defined as either low (< 25) or moderate-high ( ≥ 25) (evaluated retrospectively for postnatal women). Logistic regression was used to identify factors associated with moderate–high decisional conflict. Qualitative data were collected through focus groups with pregnant and postnatal women, and an inductive approach was used for data analysis. Among 174 pregnant and 102 postnatal women, 67.8% and 69.6%, respectively, reported moderate–high decisional conflict during pregnancy. Unsatisfactory doctor-patient relationship was associated with greater likelihood of having moderate–high decisional conflict in pregnancy, both in pregnant (aOR = 1.20, 95% CI: 1.00–1.44) and postnatal women (aOR = 1.40, 95% CI: 1.08–1.82). Reported barriers to decision-making regarding AD use in pregnancy encompassed five DCS subscales: uninformed knowledge following contradictory research and unfamiliarity with authorised resources, unclear values due to emotional blunting and fear associated with AD use, inadequate support, uncertainty in decisions and ineffective decisions due to difficulty in finding personalised treatment, and diverging recommendations by the healthcare providers (HCPs). The quality of the interaction with the HCP plays a crucial role in managing decisional conflict and supporting informed decisions in the management of perinatal mental illness. This study highlights the need for increased provision of clear, evidence-based information by HCPs to facilitate shared decision-making and create personalised treatments for perinatal women considering AD use during pregnancy.

**Supplementary Information:**

The online version contains supplementary material available at 10.1007/s00737-023-01341-0.

## Introduction

Mental illness is one of the most common morbidities of the perinatal period, which includes pregnancy and up to 1 year postpartum, affecting approximately five to 10% of women of childbearing age (Farr et al. 2014; Gavin et al. 2005). Perinatal mental illness can have negative impacts on maternal–fetal health and can lead to cognitive, emotional, and behavioural disturbances in the early mother-infant relationship (Becker et al. 2016; Biaggi et al. 2016). Given the high prevalence and significant morbidity associated with perinatal mental illness, it is important to use evidence-informed management approaches (Becker et al. 2016).

Antidepressants (ADs) are commonly used to treat perinatal mental illness, particularly depression and anxiety (Kittel-Schneider et al. 2022). The decision to use ADs during pregnancy depends on various factors, such as illness severity, treatment history, and women’s preferences. Tailored pharmacological treatment with ADs, either alone or in combination with psychotherapy, may be necessary (Dennis et al. 2017; Gavin et al. 2005; Vigod et al. 2016). However, making treatment decisions, including the use of ADs, can be challenging for healthcare providers (HCPs) and perinatal women due to limited evidence about the comparative benefits to women (Bayrampour et al. 2020). Research has primarily focused on possible medication harms to the offspring (McDonagh et al. 2014), leading to uncertainty that can impact women’s mental health and result in decisional conflict regarding the use of ADs.

Studies have shown that approximately 60% of pregnant women facing decisions about the use of ADs experience high decisional conflict (Barker et al. 2020a, b). Perceived barriers to decision-making include patient factors such as disease severity, difficulty in assessing risk–benefit, and overestimating the adverse effects of ADs, as well as institutional factors such as unavailability of psychotherapy, lack of access to high-quality information, and general practitioners' (GP) limited expertise regarding pharmacotherapy (Hippman and Balneaves 2018). However, only a few studies from the USA and Canada and one narrative review have examined this topic (Barker et al. 2020a, b; Hippman and Balneaves 2018; Patel and Wisner 2011; Walton et al. 2014a, b). One mixed-methods study showed that many pregnant women with depression reported uncertainty regarding how to treat their illness, and that those with more severe depressive symptoms were more likely to endorse decisional conflict (Walton et al. 2014a, b). No similar studies from Europe have been published, creating an important knowledge gap considering the differences in AD treatment rate between European and non-European countries (Molenaar et al. 2020) and the lack of homogenous treatment recommendations (Molenaar et al. 2018). Furthermore, the few prior studies were either conducted in clinical settings or were solely based on survey data, limiting the representativeness of the results. Therefore, further exploration of treatment decision-making difficulties in the perinatal population is warranted.

User participation and democratisation of healthcare is gaining more attention in clinical practice, and HCPs play a significant role in ensuring user participation (Stevenson et al. 2000). It is important to use evidence-based shared decision-making when considering to choose ADs, however, in the context of pregnancy or lactation, this process involves weighing the possible risk of exposure in utero or in breast milk against the potential adverse effects of sub-optimally treated maternal perinatal depression to both the mother and child. The complexity of the decision-making process around ADs in pregnancy and postpartum calls for a deeper understanding of women’s perspectives and needs to ensure that the best possible patient-centred treatment choices are made.

In this mixed-methods study, we sought to examine treatment decisional dilemma among women using or considering using AD medication during pregnancy, including quantitative and qualitative factors that govern this decision-making. In addition, we aimed to converge both the analyses for comparison and interpretation (Sandelowski 2000).

## Materials and methods

### Quantitative study phase

#### Study design and participants

We conducted a sequential mixed-methods study called HEALTHx2 where the qualitative aspect expanded upon participants’ responses in the initial quantitative phase (Pluye and Hong 2014). The theoretical underpinnings for this study were gained from Ottawa Decision Support Framework (ODSF) (O’Connor and Jacobsen 2007). The ODSF has been used as an evidence-based, practical approach to good decision support (O’Connor and Jacobsen 2007). Participants were recruited from Norway between June 2020 and June 2021. The quantitative data were collected using an electronic questionnaire administered via “Nettskjema” provided by the University of Oslo. Participants could choose to access the questionnaire anonymously or by using their national ID number. The complete questionnaire and information on recruitment methods have previously been published (Bjørndal et al. 2022). The current study included only women who participated using their national ID numbers, as only these consented to participate in the qualitative phase of the study.

In the overall study, women were eligible to participate if they (i) were in the age group 18–55 years; (ii) were planning a pregnancy, were pregnant, or had given birth within the last 5 years (hereafter, mothers); and (iii) have or previously have had a mental illness and had been offered treatment with an AD within the last 5 years. In this specific study, only pregnant and postnatal women were included.

#### Outcome

The main outcome of this study was decisional conflict, measured retrospectively in postnatal women and prospectively in pregnant women, using the Decisional Conflict Scale (DCS) (O’Connor and Jacobsen 2007) in the electronic questionnaire. Both pregnant and postnatal women were asked to report on their decision-making difficulties related to ADs in the pregnancy period (Bjørndal et al. 2022). The DCS comprises 16 items measuring five dimensions of decision-making: feeling uncertain, uninformed, unclear about values, unsupported, and ineffective decision-making. The score ranges from 0 (no decisional conflict) to 100 (extremely high decisional conflict). In line with prior research, the total DCS score was categorised into low (score < 25) and moderate to high (score ≥ 25) decisional conflict. A total score of ≥ 25 is indicative of distress as well as delayed and ineffective decision-making (O’Connor and Jacobsen 2007). The DCS has five subscales: (1) informed (3 items): how adequately informed the individual feels about the treatment options and each of their potential risks and benefits; (2) values clarity (3 items): how much difficulty an individual is having with weighing the personal importance of the potential risks and benefits of each treatment option; (3) support (3 items): perceived level of support with the decision-making process; (4) uncertainty (3 items): level of uncertainty in choosing between options; and (5) effective decision (4 items): how confident the individual is that she has made the best possible choice. The DCS has been used previously in perinatal populations (Barker et al. 2020a, b; Walton et al. 2014a, b).

#### Mental health factors and use of antidepressants

Participants were asked if they had previously received or were currently receiving psychotherapy (dichotomised as no previous or current psychotherapy received before, during, or after pregnancy). Participants could indicate whether they currently had or previously had a mental illness within a predefined list including depression, anxiety, obsessive–compulsive disorder, eating disorder, other mental illness, or no mental illness. As the diagnosis of mental illness might not have been clinically verified by the HCP, a clinical distinction between minor and major depression had not always been made, but symptomatology was rated by the use of the Edinburgh Depression Scale (EDS).

Active depressive symptoms at the time of study participation were measured using the EDS (Cox et al. 2014). The EDS is a self-rating 10-item scale validated in pregnancy and postpartum period for major and minor depression in clinical settings, with satisfactory Cronbach’s alpha reliability (0.87). The EDS has previously been validated in a Norwegian sample (Cox et al. 2014). Women are asked to rate how they have been feeling in the past 7 days. Each item response scores 0–3 on an ordinal scale, producing a total EDS score of 0–30. Higher scores indicate worse symptomatology. A cut-off score of ≥ 13 was used to determine the presence of active depressive symptoms. Symptoms of depressed mood and anhedonia over the past 2 weeks were also captured using the Patient Health Questionnaire-2 (PHQ-2) to screen for depression in a “first-step” approach (Kroenke et al. 2003). In addition to mental illnesses, the survey collected information on participants’ AD use before, during, and after pregnancy. The patterns of use were defined as non-users before or during pregnancy, continuers in pregnancy, discontinuers before pregnancy, initiators in pregnancy, and re-initiators after childbirth.

Participants were asked to indicate their perceived effectiveness of ADs for treating mental illness both in general and during pregnancy, by rating this on a scale from 0 (not at all) to 10 (very useful). To examine the risk perception of ADs (how potentially harmful they could be to the child’s development when used perinatally), participants were asked to rate it on a scale from 0 (not at all) to 10 (very harmful). This question has previously been used in another multinational study that measures women’s perception of risk to the foetus with regard to psychotropic drugs, alcohol, herbal medicines, smoking, and thalidomide (Petersen et al. 2015). To examine the relationship between the perception of benefit and the perception of risk, the benefit-risk balance was calculated. The positive benefit-risk balance indicated that the perception of benefit is higher than the perception of risk, while negative values indicated that the perception of risk is higher than the perception of utility.

Finally, the Antidepressant Compliance Questionnaire (ADCQ) scale was used to assess the doctor-patient relationship and partner support among women who reported using ADs before, during, or after their pregnancy (11). Only four items related to HCP and partner support from this 33-item scale were used in this study. Each item was rated on a 4-point scale from 1 (mostly agree) to 4 (mostly disagree) producing a total score of 4 to 12 for the relationship with the doctor, and 1 to 4 for the partner relationship. Higher scores mean that the woman is not content with her doctor and the information provided regarding treatment, and that her partner does not show a positive attitude towards her treatment with ADs.

#### Sociodemographic and maternal characteristics

These included pregnancy status at the time of study participation (i.e. currently pregnant or postnatal), maternal age, mother tongue different from Norwegian, education, marital status, parity, occupation, planned pregnancy, body mass index (BMI) at time of conception, gestational age, and smoking in pregnancy, and for those postpartum: child’s age and breastfeeding status.

### Qualitative study phase

This study adopted a focus group study design. We purposively recruited participants with moderate to high DCS score on AD use during pregnancy from three different AD exposure groups, i.e. continuers in pregnancy, discontinuers before pregnancy, and non-users. All women reported depression, alone or in combination with anxiety. Qualitative transcript-based data were collected through four semi-structured virtual focus groups to identify trends and patterns in women’s decisional conflict regarding the use of ADs. Thirteen women from across Norway participated in four virtual focus groups (Supplementary 1).

To conduct the virtual focus groups, the online platform Zoom was used. A moderator with expertise in perinatal mental health led each virtual group and used a semi-structured interview guide (Supplementary 2). A co-moderator observed each session via the internet and assisted with logistical issues. The duration of each focus group was limited to between 40 and 60 min and was performed in Norwegian. The focus groups were audio-recorded and transcribed, capturing only pertinent data for the study. During the focus groups, we sometimes experienced technical issues like audio problems and waiting for some participants. We did not include such data and pauses, stutters, and other noises in the transcripts. The Norwegian transcripts were translated into English to facilitate independent data analysis. We initially used Google translator, and thereafter two research team members corrected and verified the translations. The transcripts also underwent “denaturalisation”: a process in which “existing noises” are removed and “non-standard speeches and accents are standardised” (Oliver et al. 2005).

#### Data analysis

Descriptive quantitative statistics were conducted as appropriate. Associations between sociodemographic and maternal characteristics and moderate to high DCS scores were estimated by univariate and multivariate logistic regression models; results are presented as adjusted odds ratios (aOR) with 95% confidence intervals (CI). Candidate variables were first selected in the univariate analyses, based on *p*-value < 0.1. Then, non-significant variables (*p*-value > 0.05) and those with less than a 20% change in beta-coefficients compared to the retained variables were removed. The final adjusted models included statistically significant variables and those yielding a change equal to or greater than 20% in the beta-coefficients. Analyses were conducted separately in pregnant and postnatal women at the time of study participation, as the latter group reported retrospectively on their DCS during pregnancy. Missing data on the DCS (2.9%) and maternal factors (e.g. 5.9% in mental illnesses) were imputed using multiple imputation with chained equation (20 imputations). Because some women took ADs only in the 4 years after giving birth, we conducted a sensitivity analysis which excluded women with new-onset postpartum illnesses. Data were analysed in RStudio 4.1.2 (RStudio Team, RStudio PBC, MA, USA) and Stata 16.1 (Stata Corporation, College Station, TX, USA).

Qualitative data were analysed by two researchers using an inductive approach. Data were analysed based on the principles of systematic text condensation, which included (1) getting an overall idea by reading all the transcripts and identifying initial codes; (2) recognising initial themes based on the list of codes from each focus group; and (3) creating synthesised descriptions of the codes describing the similar phenomena under overarching concepts and themes (Malterud 2012) (Supplementary 3). The data saturation point was deemed to be reached when same themes were repeated by the participants. The software NVivo (version 12; QSR International) was used for the qualitative analysis.

Finally, to achieve convergence, the identified themes were linked with results from the quantitative analysis of the DCS subscales for comparison and interpretation (Sandelowski 2000).

#### Ethics

This study was carried out in compliance with the Helsinki Declaration. Electronic informed consent was given by each participant. The Regional Ethics Committee in Norway, region Southeast (reference number 94347), and the Norwegian Centre for Research Data (reference number 943055) approved the study. All data were stored and analysed within TSD, a service for secure data storage within the University of Oslo.

## Results

### Quantitative results

**Fig. 1:**
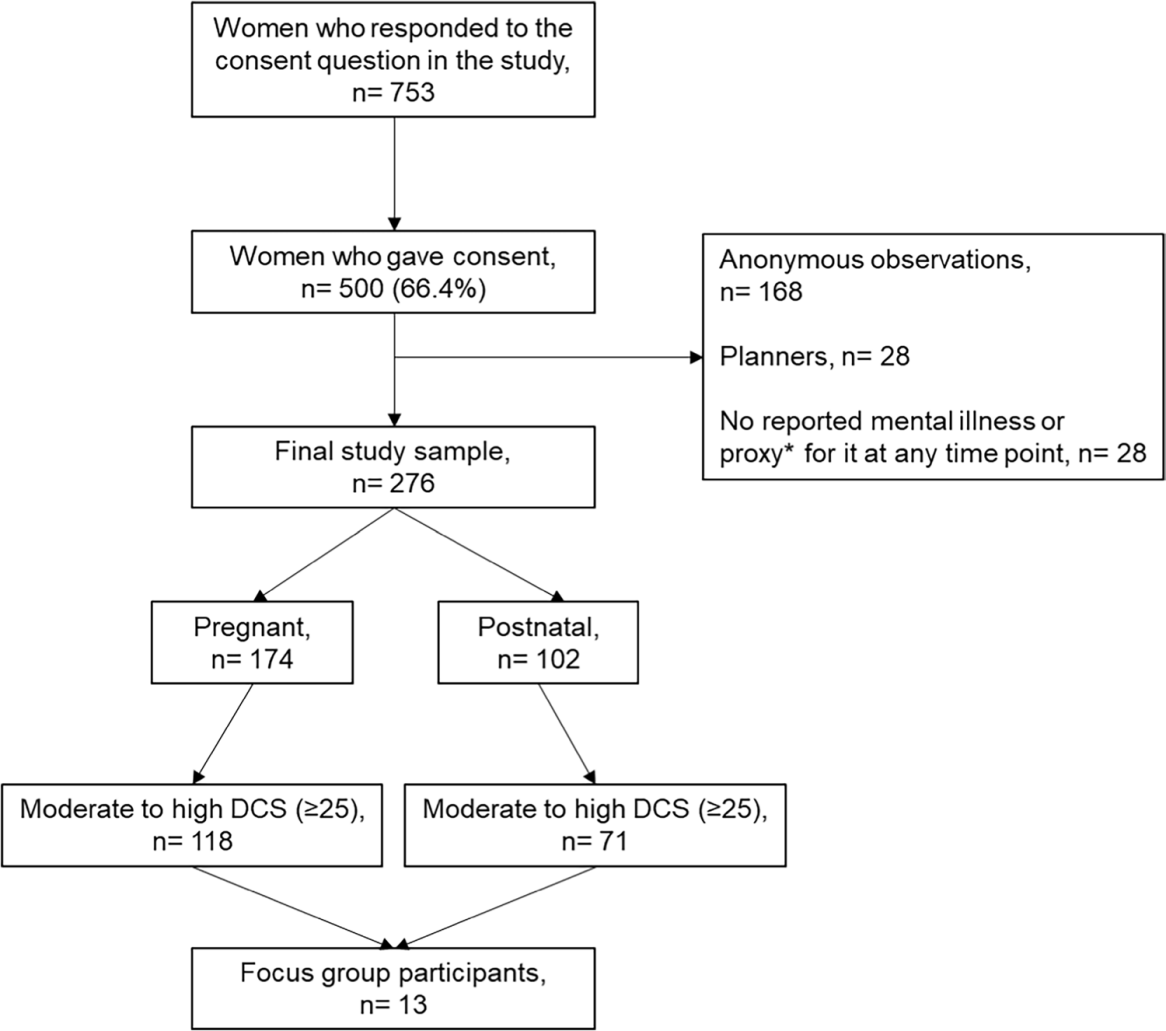
Data flow to achieve the final study sample. DCS, decisional conflict scale. *Proxies of mental illness included having used a psychotropic medication or non-pharmacological psychotherapy in the past or currently, having active depressive symptoms or self-harm thoughts at the time of questionnaire response as measured by the EDS scale (> 10) or the PHQ-2 scale (responded as “yes” to at least 1 item out of 2) or having used ADs any time before and 1 year after pregnancy. These proxies were used to verify whether women with missing or no reported mental illness based on self-reported diagnoses had proxies of mental illness (since this was an eligibility criterion in the study)

In total, 276 women reached the final study sample encompassing 174 pregnant and 102 postnatal women as summarised in Fig. 1.

Table 1 presents participants’ sociodemographic, maternal, and health-related characteristics by pregnancy status at the time of the study participation.

**Table 1:** Sociodemographic, maternal, and health-related characteristics of the study sample (*n* = 276)

	Pregnant (*n* = 174)	Postnatal (*n* = 102)
*Sociodemographic and maternal characteristics*		
Maternal age (years), mean ± SD	30.9 (4.4)	31.0 (4.7)
< 25	14 (8.1)	5 (4.9)
25–35	134 (77.0)	83 (81.4)
> 35	26 (14.9)	14 (13.7)
Mother tongue (Norwegian)	157 (90.2)	91 (90.1)
Education		
School/high school	49 (28.2)	31 (30.4)
College/university	125 (71.8)	71 (69.6)
Marital status		
Married/co-inhabiting	163 (93.7)	98 (96.1)
Single/separated/divorced/other	11 (6.3)	< 5
Occupation		
Student/homemaker	17 (9.8)	14 (13.7)
Healthcare professional	32 (18.4)	18 (17.7)
Other paid work	101 (58.0)	54 (52.9)
Unemployed/sick leave/social support	24 (13.8)	16 (15.7)
Planned pregnancy		
Yes	129 (74.1)	70 (68.6)
No	12 (6.9)	13 (12.8)
No, but it was not unexpected	32 (18.4)	19 (18.6)
BMI^a^, mean ± SD	25.8 (5.2)	25.8 (5.8)
Underweight	5 (2.9)	< 5
Normal	87 (50.0)	56 (54.9)
Overweight	82 (47.1)	45 (44.1)
Gestational age (weeks), mean ± SD	18.5 (9.9)	N/A
First trimester (< 14 weeks)	69 (39.7)	N/A
Second trimester (14 to < 28 weeks)	68 (39.1)	N/A
Third trimester (28 weeks to end of pregnancy)	37 (21.2)	N/A
Child’s age		
≤ 6 months	N/A	45 (44.1)
≥ 6 months to < 1 year	N/A	21 (20.6)
> 1 year	N/A	36 (35.3)
Breastfeeding (current or previous)	N/A	90 (88.2)
Parity		
Nulliparous	112 (64.4)	N/A
Multiparous	62 (35.6)	40 (39.2)
Smoking in pregnancy		
Yes	10 (5.8)	< 5
No	164 (94.2)	99 (97.1)
*Decisional conflict during pregnancy*		
DCS, mean ± SD	36.2 (22.1)	39.1 (22.0)
Low DCS (< 25)	46 (26.4)	28 (27.5)
Moderate to high DCS (≥ 25)	118 (67.8)	71 (69.6)
*Mental health factors and use of antidepressants*		
Mental illnesses		
Depression	139 (79.9)	94 (92.2)
Anxiety	131 (75.3)	77 (75.5)
Other mental illnesses^d^	71 (40.8)	50 (49.0)
Number of mental illnesses		
One mental illness	52 (29.9)	23 (22.5)
Two or more mental illnesses	120 (69.0)	73 (71.6)
Depressive symptoms		
PHQ-2^b^ (≥ 1), *n* (%)	100 (57.5)	58 (56.9)
EDS^c^, mean ± SD	9.3 (5.3)	10.4 (5.4)
EDS (≥ 13), *n* (%)	49 (28.2)	34 (33.3)
Preferable psychiatric treatment		
Treatment with AD^e^	25 (14.4)	7 (6.9)
Non-pharmacological treatment	53 (30.5)	42 (41.2)
Combination treatment^f^	41 (23.6)	23 (22.5)
No treatment	20 (11.5)	12 (11.8)
Unsure	34 (19.5)	17 (16.7)
Psychotherapy		
No previous or current psychotherapy	87 (50.0)	42 (41.2)
Received before, during or after pregnancy	84 (48.3)	57 (55.9)
AD use		
Non-users before or during pregnancy	50 (28.7)	28 (27.5)
Discontinuers before pregnancy	49 (28.2)	23 (22.6)
Continuers in pregnancy	67 (38.5)	34 (33.3)
Initiators in pregnancy	5 (2.9)	< 5
Reinitiators after childbirth	N/A	< 5
Preference for AD use in pregnancy		
Continue to use the same AD	38 (21.8)	19 (18.6)
Switch to another AD	7 (4.0)	< 5
Stop	81 (46.6)	40 (39.2)
Reduce the dose	20 (11.5)	10 (9.8)
No preference	27 (15.5)	27 (26.5)
Trust in safety of AD use in pregnancy		
Safe in pregnancy	103 (59.2)	54 (52.9)
Not safe	59 (33.9)	34 (33.3)
Benefit-risk perception^g^ of AD in pregnancy		
Benefit perception, mean ± SD	5.5 (3.8)	5.2 (4.2)
Risk perception, mean ± SD	4.2 (2.5)	4.5 (2.6)
Difference, mean ± SD	1.7 (5.3)	1.5 (5.7)
Benefit-risk perception of AD when breastfeeding		
Benefit perception, mean ± SD	5.5 (3.8)	5.2 (4.2)
Risk perception, mean ± SD	4.0 (2.6)	3.8 (2.9)
Difference, mean ± SD	1.7 (5.3)	2.4 (6.2)
Doctor-patient relationship^h^, mean ± SD	5.2 (2.6)	5.6 (3.0)
Partner support^i^, mean ± SD	1.8 (0.8)	2.1 (1.0)

^a^At the start of the pregnancy. ^b^*PHQ-2*, Patient Health Questionnaire-2; ^c^*EDS*, Edinburgh Depression Scale; ^d^include obsessive–compulsive disorders, eating disorders, and other mental illness; ^e^*AD*, antidepressants. ^f^Refers to pharmacological treatment together with psychotherapy; also includes unknown time period. ^g^Regarding child’s development; ^h^with regard to the doctor-patient relationship, mothers showed a higher disagreement with their doctor and information provided regarding treatment. ^i^Mothers showed a higher disagreement towards their partners not showing a positive attitude towards their treatment with ADs. Missing data in pregnant women: < 2% in planned pregnancy, preferable psychiatric treatment, psychotherapy, AD use, preference for AD use, mental illnesses, and depressive symptoms; 6.9% in belief in safety of AD use in pregnancy and 5.8% in decisional conflict. Missing data in mothers: < 2% in mother tongue, parity, preferable psychiatric treatment, preference for AD use, and depressive symptoms; 2.9% in psychotherapy, AD use; 5.9% in mental illnesses; 13.7% in belief in safety of AD use in pregnancy; and 2.9% in decisional conflict. Most postnatal women (77.5%) had a pre-existing mental illness

In total, 67.8% (*n* = 118/174) of pregnant and 69.6% (*n* = 71/102) of postnatal women reported moderate to high DCS score (≥ 25) during pregnancy. Depression and anxiety were the most common mental illnesses. Comorbidity with another mental illness was reported by 69.0% of pregnant and 71.6% of postnatal women. The majority of postnatal women (77.5%) had experienced mental illness before or during pregnancy. When asked about their preference regarding the use of ADs, stopping the AD during pregnancy was the strongest preference for both pregnant and postnatal women. Prenatal use of ADs was considered safe by 52.9% of postnatal and 59.2% of pregnant women. Postnatal women reported a low risk perception regarding treatment with ADs, hence, showing a higher benefit-risk difference. Both pregnant and postnatal women were slightly dissatisfied with their relationship to their HCPs.

**Fig. 2:**
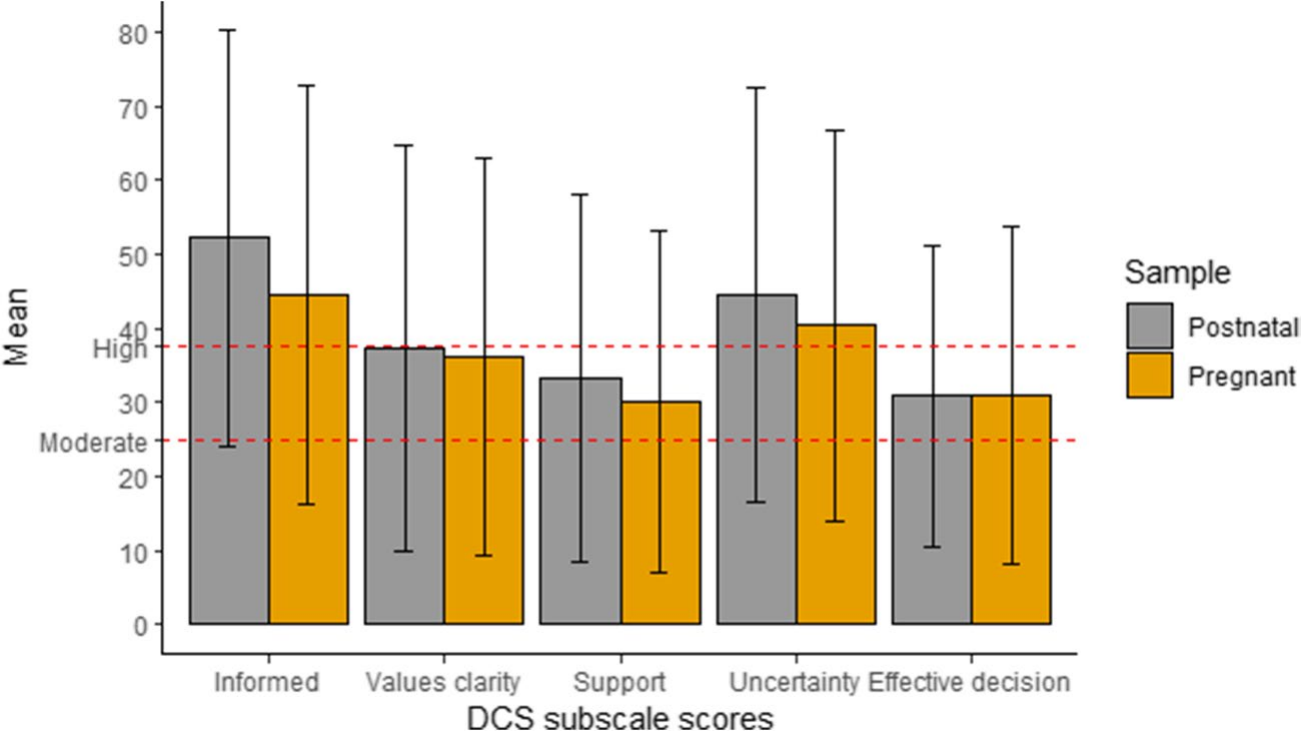
Decisional conflict scale (DCS) subscale scores among pregnant and postnatal women presented using means and standard deviations (SD)

Figure 2 presents the DCS subscale scores using means and standard deviations (SD) among pregnant and postnatal women. Both pregnant and postnatal women reported scores of ≥ 25 for all the subscales. Scores of ≥ 37.5 were observed in the “informed” and “uncertainty” subscales. The Cronbach’s alpha for the total DCS was 0.71 among pregnant and 0.75 among postnatal women.

The results of the sensitivity analysis excluding the 23 women who developed new-onset postnatal mental illness did not materially deviate from the main analysis (Supplementary 4 and 5).

### Factors associated with moderate to high-decisional conflict during pregnancy

Results of the univariate and initial full multivariable analyses between the individual maternal factors and moderate to high DCS score are shown in Supplementary 6 and 7 for the pregnant and postnatal sample, respectively. In the adjusted multivariable analysis, few factors were independently associated with moderate to high DCS score in pregnancy (Table 2). Pregnant women preferring either non-pharmacological treatment or no treatment were more likely to experience moderate to high DCS score (aOR = 2.26, 95% CI: 1.13–4.49) relative to those not having such preference. Among AD users, an unsatisfactory doctor-patient relationship was positively associated with greater likelihood of having moderate to high DCS score in pregnancy, in both pregnant (aOR = 1.20, 95% CI: 1.00–1.44) and postnatal women (aOR = 1.40, 95% CI: 1.08–1.82). No other factors were found to be significantly associated with DCS; having multiple mental illnesses was borderline associated with greater DCS score in postnatal women (Supplementary 7). Similar findings were observed in the complete case analyses, which align with the results obtained from the multiple imputed data analyses (data not shown).

**Table 2:** Results of adjusted multivariable logistic regression for the association between moderate to high DCS score in pregnancy and maternal factors

Predictors	Pregnant	Postnatal
	aOR (95% CI)	aOR (95% CI)
Preferable psychiatric treatment		
Treatment with AD/combination of psychotherapy and pharmacological treatment	Ref	NI
Non-pharmacological treatment/ No treatment/ Unsure	2.26 (1.13–4.49)	NI
Doctor-patient relationship	1.20 (1.00–1.44)	1.40 (1.08–1.82)

*aOR*, adjusted odds ratio; *NI*, not included

### Qualitative results

In total, 13 women participated in the focus groups (pregnant = 9, postnatal = 4) (Supplementary 1). The sociodemographic, maternal, and health-related characteristics of the focus group participants are presented in the supplementary materials (Supplementary 1). Table 3 presents the comparison of quantitative DCS subscale findings and qualitative data. Our analysis resulted in 11 subthemes indicating decisional conflict among the participants regarding their treatment with ADs (Supplementary 3). Women who scored higher or lower in the DCS subscales also reported similar responses in the qualitative data, hence, mutually confirming each other. We did not find any dissonance between our quantitative and qualitative results.

**Table 3:** Comparison of quantitative questions and qualitative quotations

Quantitative DCS subscales	Moderate to high DCS (≥ 25) score in pregnancy		Qualitative themes	Qualitative sub-themes	Supportive decisional conflict quotations
	Pregnant (*n* = 118), mean ± SD	Postnatal (*n* = 71), mean ± SD			
Informed	56.0 (24.0)	63.8 (21.8)	Uninformed knowledge	Contradictory research and unfamiliarity with national authorised resources	“What concerns me is that there are not many studies and research on what my AD can do over time or neurologically in a very vulnerable period of development such as pregnancy, e.g., autism or that it can damage the brain.”—P4 “When I was pregnant, I was admitted to the local psychiatric service twice because I was so depressed that I struggled to regulate my circadian rhythm and eat at the right time. Taking AD was also difficult as I did not experience good effect of it previously, and because I did not know, how it could affect the foetus. It is not enough for me when it is said that ADs are safe. I don’t know if there is enough research.”—P11 “I think if it is not dangerous to be on ADs then that should be made clear. This should be normalised and then it should be up to the individuals whether they want to use the offer or not.”—P12
Value clarity	46.9 (24.4)	48.5 (24.0)	Unclear values	Emotional blunting due to AD use	“I noticed and I don’t know if it was the medicine that did it, but in the aftermath of having used and stopped the AD, I have really struggled to find myself again and experience the normal feelings such as anger, joy, and frustration. Then one thinks that something else could happen. I don’t know”—P10 “I felt at the beginning that I had more side effects from ADs than any benefit because I experienced some suicidal tendency. It was as if you do not have ups and downs, you feel flattened”—P10
				Fear of adverse effects on child’s health	“I take it as something I need and it is not something that my child needs. I did not want to give something to my child that he doesn’t need.”—P3 “I’m a little scared that the child will get the same withdrawal symptoms as me when I tapered the dose. That I have made the child dependent on medicine because I have taken it throughout pregnancy.”—P5 “I’m waiting until week 17 to go to the specialist to see if everything is okay. I’m afraid that it might turn out that there is something wrong with the foetus and then it is my fault. I’m a little more afraid that the baby could get major developmental disabilities that make me have to have an abortion. I do not want to tell anyone until I know if things look good”—P6 “I don’t want to give my child medicine that he doesn’t need. Just want the best for the child.”—P7 “I was very worried that something might happen. That I could have a miscarriage and things like that. That’s why I decided to taper.”—P8 “I am most afraid that the child will get anxiety and depression.”—P9 “It was uncomfortable to think that I had to take a medicine that in the worst case could affect the baby.”—P10 “I experienced a lot of tiring thoughts; would I be happy about this? Would I be sorry for this because I’m on AD?—P11 “I had been informed that one should not use AD in pregnancy. I reckoned that it was because it could in some way be harmful to the foetus or you more easily miscarried. The impression I got was that you just do not go on ADs when you are pregnant.”—P12 “For me, it was the fear of what the medicine will do to my body. I felt I had no control over what would happen and what side effects it would have. I was also worried about myself becoming addicted to it.”—P12 “I was afraid of ending up in a very low mood and that I would not be there either emotionally or physically for my baby. It was a concern I had because I knew that if I ended up in a low mood, I would disconnect from the world.”—P13
Support	39.8 (21.0)	44.4 (19.8)	Inadequate support	Social pressure	“I think it was the whole package that made me choose to keep taking it during the pregnancy. We spent 3 years trying to get pregnant, first regular answers from GP, wait, everything possible: both of us undergoing blood tests; my partner was told that he was OK, then I am unsure if it's me there's something wrong with; family members who told me they were expecting us to have children.”—P10
				Mental health specialists' referral issues	“It is very scary that for your mental health you have to fight all the time, call your GP, call that person, push for something. You have to be almost completely sick to get help. If you are not terminally ill then you cannot get help. It is very scary because I think it is not you who should seek the information all the time; it is something you should just get.”—P9 “I would have liked to not use AD upfront. If I could have received more help and perhaps been taken more seriously. More holistic treatment than just being rejected and being told that one does not fulfil the criteria for specialised treatment.”—P10 “I feel that as a pregnant woman I do not really count that much. The first appointment with the midwife is to ask about e.g., mental health. I told openly about my mental health. She reacted immediately and sent referral request right away. Then a couple of weeks passed and it was rejected because I was not very ill. She was worried about how I would get through the pregnancy. It was during that time that the depression escalated. It was so hard to get help. If they had cared from the beginning, I might not have had to be hospitalised.”—P11
				Limited support and follow-ups	“I still do not know what would have been best or what the best choice is for next time. I will just stop taking the medication. I have no one to support me.”—P10 “I have been plagued with depression on and off for many years. When I started AD despite the fact that I actually had psychological help, I felt that I did not get support for how I experienced it. Therefore, when I was pregnant and became very depressed, I chose not to take an AD. In retrospect, I’m not sure if it was the right decision, and I still regret I did not start AD.”—P11
				Low level of empathy among healthcare providers	“Maybe professionals when they put on the uniform they take on the role of a doctor, maybe they should humanise a little when they act in this role. It’s about women.”—P2 “Understanding is very important because everyone has such a unique story.”—P3 “They should have asked me what are you really worried about. I know I do not know anything about it but then someone could have helped me. It is hard to get through the day. I do not have any capacity to contact online help.”—P11
Uncertainty	51.9 (22.4)	57.2 (21.5)	Uncertainty in decisions	Patient’s own uninformed decisions	“They said that you should be able to make a choice yourself and choose to stop taking the ADs but I didn’t dare because I didn’t know how it would go, so I chose to reduce the dose.”—P1 “I wanted to use a smaller dose. I felt for my own part that it was in a way a little safer but whether it was, I don’t know.” – P3 “It is a great pity that one has to struggle to find the knowledge oneself: to find out about preventive help at the beginning of the pregnancy and be able to talk to someone, to be able to make the assessment. My GP initially did not want to do anything, but I also talked to friends and several had good experiences with ADs. So then I asked him to start me on an AD, or rather I persuaded him to do it.”—P4
				Limited expertise among healthcare providers	“Both my GP and psychiatrist knew nothing about my condition or had some experience or competence for what they said. They would just read from the catalogue of medicines.”—P1 “Very good GP I have but he does not have enough competence with ADs so he thought I should quit but then I’m a trained health professional myself so I read a bit and thought it sounded a bit strange. I asked him to refer me to the hospital”—P6 “For me, it was the uncertainty then, because I asked the doctors and did not get a clear answer. I also tried to look it up but did not get a clear answer.”—P8 “I think in the beginning it was not cool to be told that there was a possibility to start AD. It would have been nice to be offered a conversation and an easy way to go to a psychologist to clear your head a little – during this phase of IVF when you could need it.”—P10
Effective decision	40.5 (20.5)	40.6 (15.6)	Ineffective decisions	Difficulty in finding personalised treatment	“I have quitted and started using AD; jumping back and forth between public and private [healthcare] for many years.”—P2 “I have been in so many rounds of medication and tapering. I’m aware of how physically demanding it is to taper and in combination with the fact that I do not know what my body wants or whether depression develops somewhat further when I have children. Maybe I will get postpartum depression. I’m terrified of it and if I can keep things fairly stable by taking medication then I considered in consultation with the doctor, that the risk of me getting sick was much higher than that the baby would get sick from that medication I took”—P5 “There is so much advice based on average [of a population] and the range of normal distribution is absolutely huge, and I would assume that applies to medicines for mental illnesses as well. The recommendation that it is not individualised maybe a little wrong for some.”—P9
				Diverging recommendations by the healthcare providers	“I have wondered a bit about the competence of the different doctors and how much experience they have or maybe they just come from different clinical points of view. I experienced that those at the women’s and children’s clinic were very concerned that my health should be taken better care of somehow and maybe the GP was a little more concerned that the child should be the focus. They see it from two different perspectives which could be fine, but as a patient, I wonder who is right.”—P6 “My GP recommended to use as little as possible and reduce the dose while, when I talked to the specialists at the hospital, they said that I should not start fooling around with medication during pregnancy and that I should continue my current dose. I think it would have been great if all the doctors said the same thing.”—P6 “I thought it was quite difficult to taper and concluded that since doctors could not give me a good answer, it was the best solution to quit.”—P8

#### Uninformed knowledge

Postnatal women were more often uninformed than pregnant women (DCS mean scores 63.8 vs. 56.0, Table 3). Due to limited research available and unfamiliarity with the national authorised online resources, most of the participants found it challenging to get the right help and evidence-based safety information. Almost all participants relied on web-based resources for information on the safety of AD use, but some found it to be overwhelming or unclear.

#### Unclear values

Postnatal women were more often unclear about personal values for benefits and risks than pregnant women. For some women, emotional numbness resulting from AD use posed a challenge in deciding whether to initiate or continue the treatment during pregnancy, leaving them uncertain about their choice. Others experienced decisional conflict due to fear and concerns about foetal exposure to ADs and the unknown risks they posed. All participants reported some degree of fear associated with AD use during pregnancy, which hindered their decision-making.

#### Inadequate support

Both pregnant and postnatal women reported feeling unsupported in their decision-making process during pregnancy either due to social pressure or due to referral and access issues. Participants reported not receiving enough peer support from trusted social platforms which ultimately made it hard to participate fully in shared decision-making about their treatment. Many participants reported that access to specialised care or regular counselling with a trained therapist was not easy. They had to either have more symptoms or had to wait for quite a long period to be referred. Some participants also reported low levels of empathy among HCPs when they wanted to talk about the different treatment options in pregnancy.

#### Uncertainty in decisions

Many participants reported being uncertain about their treatment choices either due to themselves having to make the final decision or due to their experiences of HCPs with limited expertise in the field of perinatal mental health. In many cases, participants informed that they had to initiate discussions with the providers about medication use during pregnancy and that they were left to educate themselves about the risks and benefits of the medications or trusting their intuition or "gut feelings" to make a decision.

#### Ineffective decisions

Participants found it difficult to find the personalised treatment for their illness. Almost all participants reported ineffective decision-making due to voluminous and inconsistent online information from non-certified sites and due to the differing recommendations by the HCPs. For instance, some HCPs would recommend tapering the AD dose, and others would prescribe a different AD in pregnancy. For others, the readiness of HCPs to prescribe ADs instead of taking a holistic approach, i.e. one with informed decision-making or recommending psychotherapy alone, made it harder for them to decide. For first-time AD users, initiating the treatment during pregnancy was confusing due to multiple treatment options. They also reported that the presence of multiple treatment options such as pharmacotherapy and/or psychotherapy made it hard to find the right treatment especially when the patient found her HCP to have insufficient expertise. For some participants, weighing the risks was not easy and somewhat tiring. They needed more support both online and during visits to have proper communication for making informed decisions with their HCPs regarding their fears and the potential adverse effects of ADs, and if they wanted to opt for non-pharmacological options as first choice.

In contrast, treatment response served as an enabler for some women in their decision-making. The euthymic women reported that they chose to receive maintenance pharmacotherapy during pregnancy as they did not want to lose the progress they had made before conception. This was the main reason that helped in easing their decision-making process. On the other hand, for one treatment-resistant participant, prior experience with AD ineffectiveness made decision-making regarding AD use easy. Four participants reported that it was because of the fear of postpartum depression that they wanted to continue the use of an AD during pregnancy. For some participants, the severity of the disease itself was the main enabling factor to continue using an AD.

## Discussion

This study examined shared decision-making perspectives among women using or considering to use AD medication in pregnancy, including barriers that impact the decision on whether or not to use ADs. To our knowledge, this is the first mixed-methods study to investigate decision-making difficulties in a Norwegian sample of women with mental illness at the time around pregnancy. Access to mental healthcare services and AD prescribing practices vary by country, making it crucial to understand the perspectives of women in each national setting to guide public health interventions. Using a mixed-methods approach, this study provides novel knowledge on the extent of decisional conflict about AD treatment in perinatal women, as well as on individual perspectives and lived barriers in such decision-making. An unsatisfactory relationship with the HCP was associated with an increased likelihood of moderate to high decisional conflict in pregnancy. This finding was consistent among pregnant and postnatal women, suggesting that bias due to retrospective DCS assessment for postnatal women is likely to be minimal. In women experiencing moderate to high decisional conflict about AD use in pregnancy, uninformed knowledge, unclear values, inadequate support, uncertainty in decisions, and ineffective decisions are major barriers to participation in effective shared decision-making with HCPs.

The majority of women in our sample reported experiencing moderate to high decisional conflict during pregnancy. This is not surprising as high decisional conflict about treatment has been observed even among non-pregnant patients in specialist mental healthcare (Metz et al. 2018). However, the higher degree of decisional conflict reported in our study compared to others (Patel and Wisner 2011) may be somewhat counterintuitive, given Norway’s public maternal healthcare system. Compared to previous studies, our sample of pregnant women had a range of mental illnesses, a high degree of co-morbidity in diagnosis, high level of current depressive symptoms, and higher mean DCS scores during pregnancy (Patel and Wisner 2011; Walton et al. 2014a, b). About 41.4% of our pregnant sample were continuers or initiators of AD in pregnancy, which is higher than in a previous study where 32.5% of pregnant women intended to start or continue AD use (Barker et al. 2020a, b). These findings may suggest that our sample represents a moderate to severe class of perinatal mental illness, offering novel and valuable insights into the management of this high-risk group. Additionally, the prescribing threshold for ADs during pregnancy and postpartum is higher in our sample compared to other countries (Molenaar et al. 2020; Kittel-Schneider et al. 2022). Our qualitative results support these findings, as women often struggled to obtain information regarding their treatment.

In both pregnant and postnatal women, non-pharmacological treatment was the preferred option, followed by combination treatment if necessary. This preference emerged from both quantitative and qualitative analyses, with women citing decision-making barriers such as referral to specialist psychiatrists or psychologists and access issues. Focus group results indicated that the majority of women preferred to start with non-pharmacological therapy. This finding is consistent with previous studies investigating women’s anxiety or depression-related treatment preferences. Perinatal women reported psychotherapy or combination therapy as the preferred psychiatric treatment (Patel and Wisner 2011). Another Norwegian study found that pregnant and postpartum women were less likely to replace AD discontinuation with psychotherapy and psychiatric follow-up (Trinh et al. 2022). This could be due to the difficulties in accessing psychotherapy.

Although more than half of our sample believed ADs to be safe in pregnancy and breastfeeding, both pregnant (46.6%) and postnatal women (39.2%) preferred to stop AD use in pregnancy. Notably, most of the postnatal women were breastfeeding or had breastfed their infant, which possibly explains the moderately high preference towards stopping AD use before giving birth. Our observed risk–benefit perception in pregnancy and during breastfeeding indicates that women rated the benefit of ADs to slightly outweigh possible risks to the offspring. This discrepancy in safety perception and willingness to use ADs, alongside ADs not being the preferred treatment, is further evident by subthemes such as emotional blunting and fear of adverse effects due to AD use. Researchers in one study of pregnant women in Canada similarly reported that women found it difficult to decide whether to use ADs due to the uncertainty regarding their impact on the foetus (Walton et al. 2014a, b). Hence, such results reinforce that use of ADs in the perinatal period is still considered to carry risk even though the positive risk–benefit profile of SSRIs in the perinatal period is widely recognised now (Spigset and Nordeng 2016). These findings also lend additional credence to the notion that the extent to which new evidence-based knowledge about the general safety profile of ADs is divulged to HCPs, peers, and women, and integrated into clinical practice guidelines, is unclear (Kittel-Schneider et al. 2022). It is important to recognise that discontinuation of ADs during pregnancy has been associated with an increased risk of acute relapse in mental state (Bayrampour et al., 2020) and not all women can therefore safely discontinue their ADs once pregnant. One important factor that emerged in the ineffective decisions subtheme is that past treatment response to ADs plays a crucial role in the decision-making, in addition to disease severity. This is novel and clinically relevant finding that needs to be implemented in clinical practice guidelines.

One key finding is that an unsatisfactory relationship with the HCP was associated with an increased likelihood of moderate to high decisional conflict about ADs in pregnancy. In the focus groups, women cited low levels of empathy and limited expertise among HCPs as causes of decisional conflict. Rapport with relevant HCPs has previously been discussed as an important factor in decision-making regarding the use of psychotropic medication (Barker et al. 2020a, b; Stevenson et al. 2016). In addition, women reported that HCPs often recommended contradictory treatment options. Previous studies have shown that HCPs may be reluctant to recommend or may have conflicting opinions regarding psychotropic medication use in pregnancy and during breastfeeding, due to insufficient safety data (Bilszta et al. 2011; Ververs et al. 2009). This implies that shared decision-making together with evidence-based practice is still limited in the context of treating perinatal mental illness.

Women reported having to deal with either a plethora of web-based conflicting information or limited research regarding the use of ADs in pregnancy. From the HCPs’ perspective, there are no harmonised or up-to-date pharmacological management guidelines to guide clinical decision-making about perinatal depression in Europe (Kittel-Schneider et al. 2022). However, in Norway, medicine information centres like RELIS (“RELIS—Produsentuavhengig legemiddelinformasjon for helsepersonell”, 2022) and the public platforms like Tryggmammamedisin (“Tryggmammamedisin”, 2022) provide up-to-date, evidence-based information. Likewise, ENTIS in Europe (“European Network of Teratology Information Services (ENTIS)”, 2022), UKTIS in the UK (“UK Teratology Information Service (UKTIS)”, 2022), and MotherToBaby in North America (“MotherToBaby”, 2022) are also eminent resources for both HCPs and patients. Efforts should be made to improve dissemination of information about the existence of such resources to GPs, midwives, and women of childbearing age.

Our study has many strengths. We were able to capture a large study sample, namely women with a mental illness around the time of pregnancy, from all regions of Norway. The qualitative part of the study even took place during the unprecedented scenario of the COVID-19 pandemic. It provides a deeper understanding of their barriers to decisional conflict through qualitative focus groups. The qualitative findings conceptually corroborated the DCS subscales, hence, pointing towards high decisional conflict among our sample. We collected a variety of themes and reached data saturation to achieve the study aim. Finally, we were able to recruit women from a range of settings which provided a diverse range of experiences and severity of illness within the sample. The study used screening tools and diagnostic algorithms validated and/or used in prior research in Norway (Cox et al. 2014; Kroenke et al. 2003; Petersen et al. 2015).

The study has limitations that need to be addressed. Women reported their preference at one point in time which does not account for the fact that preferences change over time and that decision-making is time sensitive. Women were eligible to participate up to 5 years postpartum, which may affect accurate recall of past experiences during pregnancy. Postnatal women having children older than one year of age may no longer face decisional conflict about AD treatment in pregnancy or while breastfeeding, and this may affect their recall about the pregnancy period. However, our study included only a small proportion of women with this characteristic and the DCS questions related specifically to the time around pregnancy (Bjørndal et al. 2022). To further address this issue, we conducted all the analyses separately in pregnant and postnatal women, and results were consistent in both the groups. Yet, we cannot exclude the possibility that postnatal women having a more favourable mental health status and not taking ADs at the time of study participation may recall past decision-making difficulties differently than their counterparts. However, we observed no major differences on the DCS and its subscales between pregnant and postnatal women. Our findings may not be generalisable due to the sample being composed of predominantly well-educated and employed women. Besides, we did not collect race and ethnicity data which could have provided information on disproportionate differences across populations. Additionally, due to the sequential nature of the study design, we cannot ignore potential bias. We could not calculate the conventional response rate due to the use of an electronic questionnaire and several recruitment strategies. However, among the women who expressed their willingness to participate in the study, the response rate was satisfactory (66%). The validity of web-based recruitment methods is now well acknowledged (Statistics Norway, n.d; van Gelder et al. 2010), and the internet penetration rate is almost 100% in women of childbearing age in Norway. We cannot exclude the possibility that the women who decided to participate in the study differed from the general population of perinatal women with mental illnesses in ways that our analysis could not control for. During the focus group recruitment, the dropout was high which might suggest that some women were either too mentally unwell or uncomfortable with virtual interaction or needed to organise for childcare during the meetings. During the videoconferencing, more prompting was needed to involve the participants in the discussion among themselves. Lastly, the study was limited to Norwegian-speaking participants. The DCS is not validated in Norwegian and is not specific to the perinatal period. However, the scale was translated and back-translated by two independent linguistic experts and it has been used in perinatal settings (Barker et al. 2020a, b; Walton et al. 2014a, b).

## Conclusion

Among several barriers to decision-making, one of the most significant was the quality of interaction between HCPs and patients. Many perinatal patients reported feeling dissatisfied with the level of interaction and support they received from their HCPs, which hindered their ability to make informed decisions about managing their mental illness during pregnancy. Women with a mental illness during pregnancy require access to up-to-date and accurate information about the safety of ADs, but they often find this information difficult to obtain from their HCPs. Women expressed a desire to participate in shared decision-making and to receive support in exploring all available options to make informed decisions that align with their preferences. Therefore, our study highlights the need for HCPs to offer comprehensive and evidence-based information to facilitate shared decision-making and the development of personalised treatment plans for women who require ADs during pregnancy.

## Supplementary Information

Below is the link to the electronic supplementary material.

Supplementary file1 (DOCX 290 KB)

## Data Availability

All data relevant to the study are included in the article or provided as supplementary information. Researchers have the opportunity to request data access for subprojects aligned with the primary objectives of the main study ‘HEALTHx2’.

## Abbreviations

AD: Antidepressants
ADCQ: Antidepressant Compliance Questionnaire
DCS: Decisional Conflict Scale
EDS: Edinburgh Depression Scale
GP: General practitioner
HCP: Healthcare provider
PHQ-2: Patient Health Questionnaire-2

## References

[CR1] Barker LC, Dennis C-L, Hussain-Shamsy N, Stewart DE, Grigoriadis S, Metcalfe K., . . . Vigod SN (2020a). Decision-making about antidepressant medication use in pregnancy: a comparison between women making the decision in the preconception period versus in pregnancy. BMC psychiatry, 20(1), 54–54. 10.1186/s12888-020-2478-8 10.1186/s12888-020-2478-8 PMC7007680 32033547

[CR2] Barker LC, Dennis CL, Hussain-Shamsy N, Stewart DE, Grigoriadis S, Metcalfe K., . . . Vigod SN. (2020b). Decision-making about antidepressant medication use in pregnancy: a comparison between women making the decision in the preconception period versus in pregnancy. BMC psychiatry, 20(1). 10.1186/s12888-020-2478-8 10.1186/s12888-020-2478-8 PMC7007680 32033547

[CR3] Bayrampour H, Kapoor A, Bunka M, Ryan D (2020). The risk of relapse of depression during pregnancy after discontinuation of antidepressants: a systematic review and meta-analysis. J Clin Psychiatry..

[CR4] Becker M, Weinberger T, Chandy A, Schmukler S (2016). Depression during pregnancy and postpartum. Curr Psychiatry Rep.

[CR5] Biaggi A, Conroy S, Pawlby S, Pariante CM (2016). Identifying the women at risk of antenatal anxiety and depression: a systematic review. J Affect Disord.

[CR6] Bilszta JL, Tsuchiya S, Han K, Buist AE, Einarson A (2011). Primary care physician’s attitudes and practices regarding antidepressant use during pregnancy: a survey of two countries. Arch Womens Ment Health.

[CR7] Bjørndal LD, Tauqeer F, Heiervang KS, Clausen HK, Heitmann K, Lupattelli A (2022). Perceived risk of neurodevelopmental outcomes in offspring related to psychotropic and mental illness exposures in pregnancy and breastfeeding: a cross-sectional survey of women with past or current mental illness. BMJ open.

[CR8] Cox JL, Holden J, & Henshaw C (2014). Perinatal mental health: the Edinburgh postnatal depression scale (EPDS) manual: RCPsych publications London

[CR9] Dennis CL, Falah-Hassani K, Shiri R (2017). Prevalence of antenatal and postnatal anxiety: systematic review and meta-analysis. Br J Psychiatry.

[CR10] European Network of Teratology Information Services (ENTIS). (2022). Retrieved from https://www.entis-org.eu/about

[CR11] Farr SL, Dietz PM, O'Hara MW, Burley K, Ko JY (2014). Postpartum anxiety and comorbid depression in a population-based sample of women. J Womens Health.

[CR12] Gavin NI, Gaynes BN, Lohr KN, Meltzer-Brody S, Gartlehner G, Swinson T (2005). Perinatal depression: a systematic review of prevalence and incidence. Obstet Gynecol.

[CR13] Hippman C, Balneaves LG (2018). Women’s decision making about antidepressant use during pregnancy: A narrative review. Depress Anxiety.

[CR14] Kittel-Schneider S, Felice E, Buhagiar R, Lambregtse-van den Berg M, Wilson CA, Banjac Baljak V, . . . Lupattelli A (2022). Treatment of peripartum depression with antidepressants and other psychotropic medications: a synthesis of clinical practice guidelines in Europe. International Journal of Environmental Research and Public Health, 19(4), 1973. Retrieved from https://www.mdpi.com/1660-4601/19/4/1973 10.3390/ijerph19041973 PMC8872607 35206159

[CR15] Kroenke K, Spitzer RL, Williams JB (2003). The Patient Health Questionnaire-2: validity of a two-item depression screener. Medical care, 1284–1292 10.1097/01.MLR.0000093487.78664.3C 14583691

[CR16] Malterud K (2012). Systematic text condensation: a strategy for qualitative analysis. Scand J Public Health.

[CR17] McDonagh M, Matthews A, Phillipi C, Romm J, Peterson K, Thakurta S, & Guise JM (2014). Antidepressant treatment of depression during pregnancy and the postpartum period. Evid Rep Technol Assess (Full Rep)(216), 1–308. 10.23970/ahrqepcerta216 10.23970/AHRQEPCERTA216 30313002

[CR18] Metz MJ, Veerbeek MA, van der Feltz-Cornelis CM, de Beurs E, Beekman ATF (2018). Decisional conflict in mental health care: a cross-sectional study. Soc Psychiatry Psychiatr Epidemiol.

[CR19] Molenaar NM, Kamperman AM, Boyce P, Bergink V (2018). Guidelines on treatment of perinatal depression with antidepressants: an international review. Aust N Z J Psychiatry.

[CR20] Molenaar NM, Bais B, Lambregtse-van den Berg MP, Mulder CL, Howell EA, Fox NS, . . . Kamperman AM (2020). The international prevalence of antidepressant use before, during, and after pregnancy: a systematic review and meta-analysis of timing, type of prescriptions and geographical variability. J Affect Disord, 264, 82–89. 10.1016/j.jad.2019.12.014 10.1016/j.jad.2019.12.014 31846905

[CR21] MotherToBaby. (2022). Retrieved from https://mothertobaby.org/

[CR22] O’Connor A, Jacobsen M (2007). Decisional conflict: Supporting people experiencing uncertainty about options affecting their health.

[CR23] Oliver DG, Serovich JM, Mason TL (2005). Constraints and opportunities with interview transcription: towards reflection in qualitative research. Soc Forces.

[CR24] Patel SR, Wisner KL (2011). Decision making for depression treatment during pregnancy and the postpartum period. Depress Anxiety.

[CR25] Petersen I, McCrea RL, Lupattelli A, Nordeng H (2015). Women’s perception of risks of adverse fetal pregnancy outcomes: a large-scale multinational survey. BMJ Open.

[CR26] Pluye P, Hong QN (2014). Combining the Power of stories and the power of numbers: mixed methods research and mixed studies reviews. Annu Rev Public Health.

[CR27] RELIS - Produsentuavhengig legemiddelinformasjon for helsepersonell (2022). Retrieved from https://relis.no/

[CR28] Sandelowski M (2000). Focus on research methods combining qualitative and quantitative sampling, data collection, and analysis techniques. Res Nurs Health.

[CR29] Spigset O, & Nordeng H (2016). Safety of psychotropic drugs in pregnancy and breastfeeding. Pharmacovigilance Psychiatry, 299–319

[CR30] Statistics Norway n.d. ICT usage in households. Retrieved from https://www.ssb.no/en/teknologi-og-innovasjon/informasjons-og-kommunikasjonsteknologi-ikt/statistikk/bruk-av-ikt-i-husholdningene

[CR31] Stevenson FA, Barry CA, Britten N, Barber N, Bradley CP (2000). Doctor–patient communication about drugs: the evidence for shared decision making. Soc Sci Med.

[CR32] Stevenson F, Hamilton S, Pinfold V, Walker C, Dare CRJ, Kaur H, . . . Petersen I (2016). Decisions about the use of psychotropic medication during pregnancy: a qualitative study. BMJ open, 6(1), e010130. 10.1136/bmjopen-2015-010130 10.1136/bmjopen-2015-010130 PMC4735167 26817641

[CR33] Trinh NTH, Nordeng HME, Bandoli G, Eberhard-Gran M, Lupattelli A (2022). Antidepressant and mental health care utilization in pregnant women with depression and/or anxiety: an interrupted time-series analysis. J Affect Disord.

[CR34] Tryggmammamedisin. (2022). Retrieved from https://tryggmammamedisin.no/

[CR35] UK Teratology Information Service (UKTIS). (2022). Retrieved from http://www.uktis.org/

[CR36] van Gelder MM, Bretveld RW, Roeleveld N (2010). Web-based questionnaires: the future in epidemiology?. Am J Epidemiol.

[CR37] Ververs T, van Dijk L, Yousofi S, Schobben F, Visser GHA (2009). Depression during pregnancy: views on antidepressant use and information sources of general practitioners and pharmacists. BMC Health Serv Res.

[CR38] Vigod SN, Wilson CA, & Howard LM (2016). Depression in pregnancy. BMJ (Clinical research ed.),* 352*, i1547. 10.1136/bmj.i1547 10.1136/bmj.i1547 27013603

[CR39] Walton GD, Ross LE, Stewart DE, Grigoriadis S, Dennis C-L, Vigod S (2014). Decisional conflict among women considering antidepressant medication use in pregnancy. Arch Womens Ment Health.

[CR40] Walton GD, Ross LE, Stewart DE, Grigoriadis S, Dennis CL, Vigod S (2014). Decisional conflict among women considering antidepressant medication use in pregnancy. Arch Womens Mental Health.

