# Supplementary material for: Assessing decisional conflict and challenges in decision-making among perinatal women using or considering using antidepressants during pregnancy—a mixed-methods study
Source: Arch Womens Ment Health. 2023 Jul 22;26(5):669–83. doi: 10.1007/s00737-023-01341-0 (PMC10491504; doi:10.1007/s00737-023-01341-0)
Supplement: Supplementary file 1 — Supplementary file1 (DOCX 290 KB) [file 737_2023_1341_MOESM1_ESM.docx]

**Supplementary 1**: FG participants’ characteristics

FG participants’ pregnancy status and pattern of AD use during pregnancy and postpartum^¶^

| Participants | Status | AD^*^ use | Breastfeeding |
| --- | --- | --- | --- |
| 1-3 | Pregnant | Continuer in pregnancy | N/A |
| 4 | Pregnant | Continuer in pregnancy with a maintenance dose; euthymic | N/A |
| 5 | Pregnant | Continuer in pregnancy with tapering; euthymic | N/A |
| 6 | Pregnant | Continuer in pregnancy | N/A |
| 7 | Pregnant | Continuer in pregnancy with tapering; euthymic | N/A |
| 8 | Pregnant | Discontinued in pregnancy in week 8 | N/A |
| 9 | Pregnant | Discontinued 2 months before pregnancy | N/A |
| 10 | Postnatal | Discontinued after birth; continued in pregnancy | Yes |
| 11 | Postnatal | Discontinued in pregnancy and postpartum | Yes |
| 12 | Postnatal | Never user, offered AD use in pregnancy; follow-up care from midwife | Yes |
| 13 | Postnatal | Prior user of AD, discontinued in pregnancy & postpartum; follow-up care | Yes |

^¶^One postnatal woman had delivered her child in the past 15 months, and the remainder in the past 12 months. *AD = antidepressants; N/A = not applicable

Sociodemographic, maternal, and health-related characteristics of the focus group participants (n= 13)

|  | FG^a^ participants  (n= 13) |
| --- | --- |
| *Sociodemographic and maternal characteristics* | |
| Maternal age (years), Mean ± SD | 28.4 (3.6) |
| Mother-tongue (Norwegian) | 11 (84.6) |
| Education (College/University) | 11 (84.6) |
| Marital status (Married/co-inhabiting) | 13 (100.0) |
| Occupation (Healthcare professional) | 5 (38.5) |
| Planned pregnancy (Yes) | 10 (76.9) |
| BMI^a^, Mean ± SD | 25.6 (4.8) |
| Smoking in pregnancy (No) | 13 (100.0) |
| *Mental health factors and use of antidepressants* | |
| Number of mental illnesses (Two or more mental illnesses) | 10 (76.9) |
| Depressive symptoms, PHQ-2^b^ (≥1), n (%) | 5 (38.5) |
| Depressive symptoms, EDS^c^, Mean ± SD | 8.1 (4.7) |
| Preferable psychiatric treatment (Non-pharmacological treatment) | 6 (46.1) |
| Psychotherapy (No previous or current psychotherapy) | 6 (46.1) |
| Preference for AD^d^ use in pregnancy (Stop) | 8 (61.4) |
| Trust in safety of AD use in pregnancy (Not safe) | 6 (46.1) |
| Doctor-patient relationship^h^, Mean ± SD | 6.1 (2.9) |
| Partner support^i^, Mean ± SD | 2.4 (1.0) |

^a^FG = Focus group; ^b^PHQ-2 = Patient Health Questionnaire-2; ^c^EDS = Edinburgh Depression Scale; ^d^AD = antidepressants

**Supplementary 2**: Focus group interview guide for patient-centered approaches for studying the effectiveness of antidepressants in perinatal women with depression and/or anxiety

| **Welcome** |
| --- |
| Introduction of the moderator and assistant |
| **Introduction to the study** |
| 1. Overview of the study |
| 1. In this focus group, we are interested in hearing from you about your views and experiences on most important reproductive risks of perinatal antidepressant exposure and health outcomes that matter to you. In addition, the factors related to antidepressant treatment discontinuation in pregnancy. You were recruited because you are fully representative of depression and/or anxiety. There are no wrong answers but rather differing points of view. |
| **Open-ended questions** |
| Treatment options for depression |
| 1. To start, tell us a little bit about what kind of mental health care are you seeking during have sought during pregnancy and post-partum? |
| 1. Have you received any other recommendations for treatment prior to being recommended antidepressant medication? |
| Thoughts/concerns/experiences regarding pharmacotherapy of depression, anxiety, obsessive-compulsive disorder and eating disorder |
| 1. What were your initial thoughts and feelings about the recommendation? |
| 1. Which reproductive risks and health outcomes of perinatal antidepressant exposure concern you? |
| 1. What factors motivated you to not take your antidepressant medication in pregnancy/post-partum? What factors motivated you to take your medication in pregnancy/ post-partum? |
| 1. What are the most important factors in this decision-making process for you? |
| 1. How you have gone about weighing the risks and benefits of your decision of not taking/discontinuing antidepressants? |
| 1. Tell us about disappointments or positive experiences you have had with discontinuing/not taking antidepressants? |
| **Closing** |
| 1. Any advice you would give to other women who have to make this same decision of taking or not taking antidepressants? |
| 1. Any advice to healthcare providers who may be helping their patients make this decision? |
| 1. After the brief oral summary: Is this an adequate summary? |
| 1. Of all the things we discussed, what do you think is the most important? |

**Supplementary 3**: Representation of the systematic text condensation, with initial themes (top), subthemes (middle), and final themes (bottom).


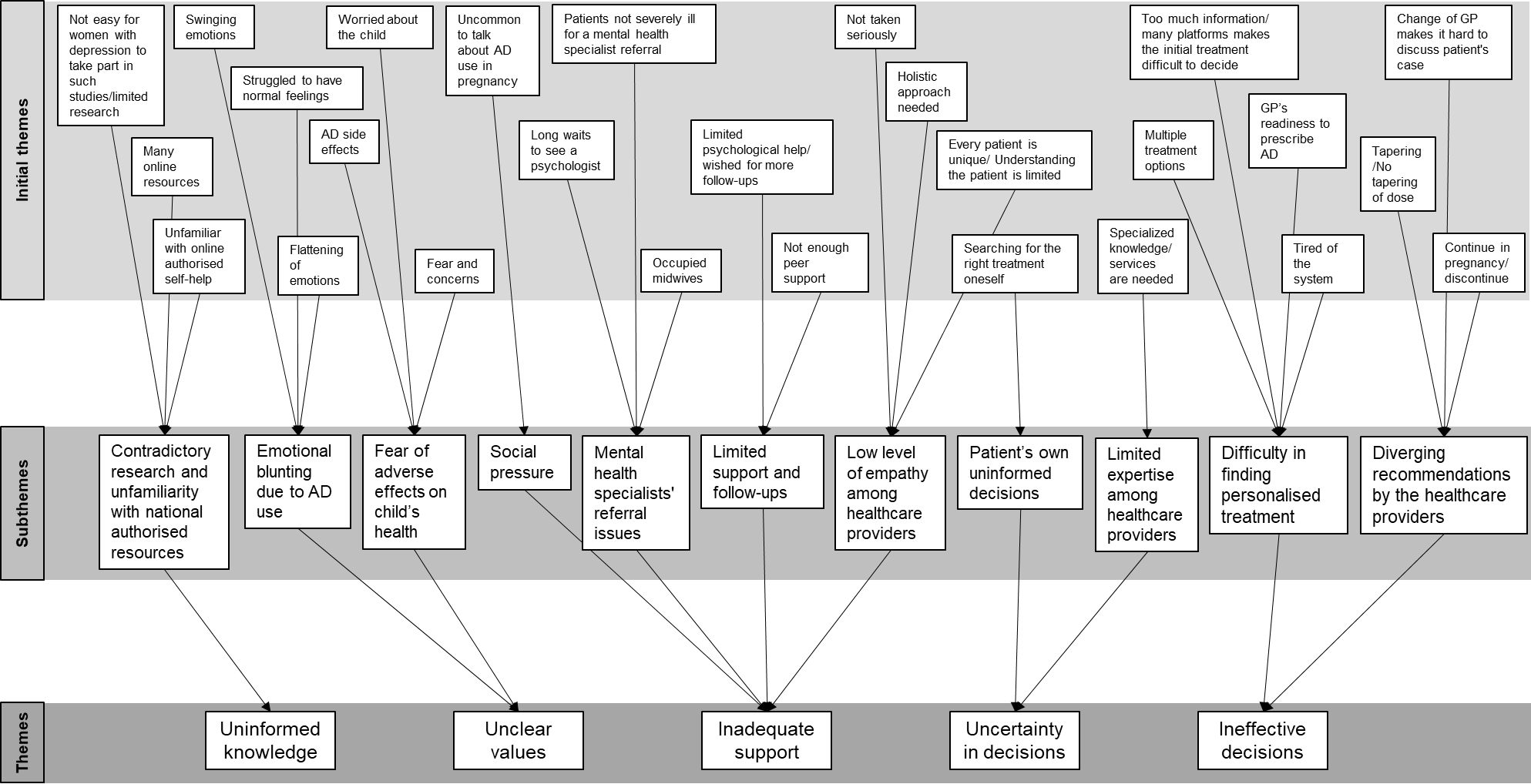


**Supplementary 4**: Sociodemographic, maternal, and health-related characteristics of the study sample excluding the 23 postnatal women having new-onset mental illness after birth (n= 253).

|  | Pregnant  (n= 174) | Postnatal  (n= 79) |
| --- | --- | --- |
| *Sociodemographic and maternal characteristics* | | |
| Maternal age (years), Mean ± SD | 30.9 (4.4) | 31.0 (4.7) |
| <25 | 14 (8.1) | 4 (5.1) |
| 25-35 | 134 (77.0) | 63 (79.7) |
| >35 | 26 (14.9) | 12 (15.2) |
| Mother-tongue (Norwegian) | 157 (90.2) | 70 (88.6) |
| Education |  |  |
| School/High school | 49 (28.2) | 24 (30.4) |
| College/University | 125 (71.8) | 55 (69.6) |
| Marital status |  |  |
| Married/co-inhabiting | 163 (93.7) | 76 (96.2) |
| Single/separated/divorced/other | 11 (6.3) | <5 |
| Occupation |  |  |
| Student/homemaker | 17 (9.8) | 12 (15.2) |
| Healthcare professional | 32 (18.4) | 14 (17.7) |
| Other paid work | 101 (58.0) | 44 (55.7) |
| Unemployed/Sick leave/Social support | 24 (13.8) | 9 (11.4) |
| Planned pregnancy |  |  |
| Yes | 129 (74.1) | 58 (73.4) |
| No | 12 (6.9) | 15 (19.0) |
| No, but it was not unexpected | 32 (18.4) | 6 (7.6) |
| BMI^a^, Mean ± SD | 25.8 (5.2) |  |
| Underweight | 5 (2.9) | <5 |
| Normal | 87 (50.0) | 44 (55.7) |
| Overweight | 82 (47.1) | 34 (43.0) |
| Gestational age (weeks), Mean ± SD | 18.5 (9.9) | N/A |
| First trimester (<14 weeks) | 69 (39.7) | N/A |
| Second trimester (14 - <28 weeks) | 68 (39.1) | N/A |
| Third trimester (28 weeks - end of pregnancy ) | 37 (21.2) | N/A |
| Child’s age |  |  |
| ≤6 months | N/A | 33 (41.8) |
| ≥6 months - < 1 year | N/A | 14 (17.7) |
| >1 year | N/A | 32 (40.5) |
| Breastfeeding (current or previous) | N/A | 70 (88.6) |
| Parity |  |  |
| Nulliparous | 112 (64.4) | N/A |
| Multiparous | 62 (35.6) | 33 (41.8) |
| Smoking in pregnancy |  |  |
| Yes | 10 (5.8) | <5 |
| No | 164 (94.2) | 78 (98.7) |
| *Decisional conflict during pregnancy* | | |
| DCS, Mean ± SD | 36.2 (22.1) | 41.3 (21.5) |
| Low DCS (<25) | 46 (26.4) | 19 (24.0) |
| Moderate to High DCS (≥25) | 118 (67.8) | 57 (72.1) |
| *Mental health factors and use of antidepressants* | | |
| Mental illnesses |  |  |
| Depression | 139 (79.9) | 71 (89.9) |
| Anxiety | 131 (75.3) | 57 (72.1) |
| Other mental illnesses^d^ | 71 (40.8) | 36 (45.6) |
| Number of mental illnesses |  |  |
| One mental illness | 52 (29.9) | 19 (24.0) |
| Two or more mental illnesses | 120 (69.0) | 54 (68.3) |
| Depressive symptoms |  |  |
| PHQ-2^b^ (≥1), n (%) | 100 (57.5) | 45 (57.0) |
| EDS^c^, Mean ± SD | 9.3 (5.3) | 10.9 (5.2) |
| EDS (≥13), n (%) | 49 (28.2) | 29 (36.7) |
| Preferable psychiatric treatment |  |  |
| Treatment with AD^e^ | 25 (14.4) | 7 (6.9) |
| Non-pharmacological treatment | 53 (30.5) | 42 (41.2) |
| Combination treatment^f^ | 41 (23.6) | 23 (22.5) |
| No treatment | 20 (11.5) | 12 (11.8) |
| Unsure | 34 (19.5) | 17 (16.7) |
| Psychotherapy | 27 (57.4) | 14 (48.3) |
| No previous or current psychotherapy | 87 (50.0) | 33 (41.8) |
| Received before, during or after pregnancy | 84 (48.3) | 45 (57.0) |
| AD use |  |  |
| Non-users before or during pregnancy | 50 (28.7) | 28 (35.4) |
| Discontinuers before pregnancy | 49 (28.2) | N/A |
| Continuers in pregnancy | 67 (38.5) | 34 (43.0) |
| Initiators in pregnancy | 5 (2.9) | <5 |
| Reinitiators after childbirth | N/A | <5 |
| Preference for AD use in pregnancy |  |  |
| Continue to use the same AD | 38 (21.8) | 17 (21.5) |
| Switch to another AD | 7 (4.0) | <5 |
| Stop | 81 (46.6) | 21 (26.6) |
| Reduce the dose | 20 (11.5) | 10 (12.7) |
| No preference | 27 (15.5) | 25 (31.6) |
| Trust in safety of AD use in pregnancy |  |  |
| Safe in pregnancy | 103 (59.2) | 46 (58.2) |
| Not safe | 59 (33.9) | 24 (30.4) |
| Benefit-risk perception^g^ of AD in pregnancy |  |  |
| Benefit perception, Mean ± SD | 5.5 (3.8) | 6.0 (3.9) |
| Risk perception, Mean ± SD | 4.2 (2.5) | 4.2 (2.8) |
| Difference, Mean ± SD | 1.7 (5.3) | 3.9 (5.5) |
| Benefit-risk perception of AD when breastfeeding |  |  |
| Benefit perception, Mean ± SD | 5.5 (3.8) | 6.0 (3.9) |
| Risk perception, Mean ± SD | 4.0 (2.6) | 3.4 (2.8) |
| Difference, Mean ± SD | 1.7 (5.3) | 3.9 (5.5) |
| Doctor-patient relationship^h^, Mean ± SD | 5.2 (2.6) | 5.4 (2.9) |
| Partner support^i^, Mean ± SD | 1.8 (0.8) | 2.1 (1.0) |

^a^ at the start of the pregnancy. ^b^PHQ-2 = Patient Health Questionnaire-2; ^c^EDS = Edinburgh Depression Scale; ^d^include obsessive-compulsive disorders, eating disorders and other mental illness; ^e^AD = antidepressants. ^f^refers to pharmacological treatment together with psychotherapy; also includes unknown time period. ^g^Regarding child’s development; ^h^With regards to the doctor-patient relationship, mothers showed a higher disagreement with their doctor and information provided regarding treatment. ^i^Mothers showed a higher disagreement towards their partners not showing a positive attitude towards their treatment with ADs. Missing data in pregnant women: <2% in planned pregnancy, preferable psychiatric treatment, psychotherapy, AD use, preference for AD use, mental illnesses, and depressive symptoms; 6.9% in belief in safety of AD use in pregnancy and 5.8% in decisional conflict. Missing data in mothers: <2% in mother-tongue, preferable psychiatric treatment; 2.5% in preference for AD use; 1.3% in psychotherapy; 12.7% in AD use; 5.9% in mental illnesses; 11.4% in belief in safety of AD use in pregnancy, and 3.8% in decisional conflict. Most postnatal women (77.5%) had a pre-existing mental illness.

**
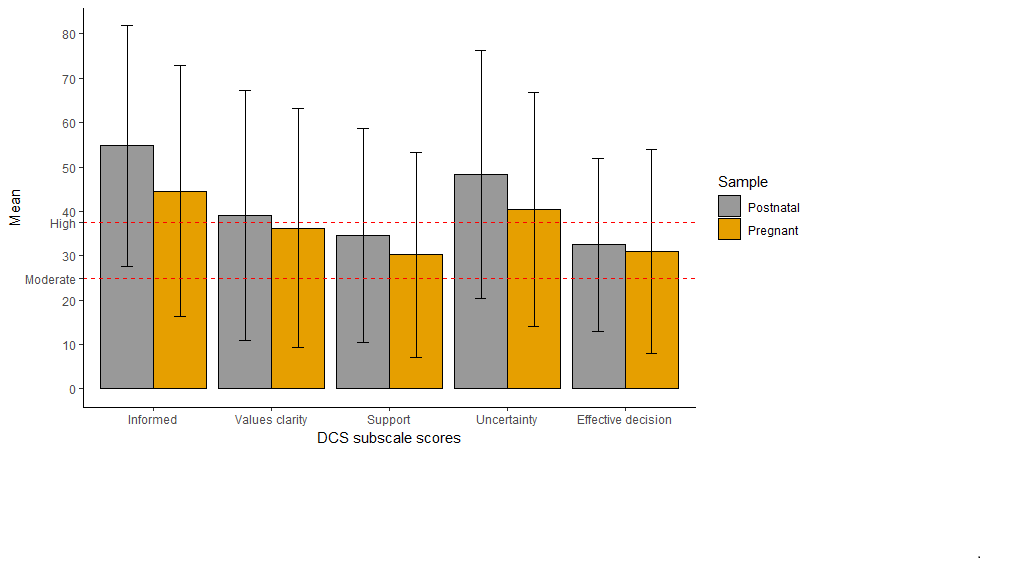
Supplementary 5** Decisional conflict scale (DCS) subscale scores among pregnant and postnatal women excluding the 23 postnatal women having new-onset mental illness after birth presented using means and standard deviations (SD)

**Supplementary 6**: Results of the univariate and the full multivariable logistic regression for the association between moderate to high DCS and maternal factors in the pregnant sample

| Maternal factor |  | | |
| --- | --- | --- | --- |
|  | **Crude OR**  **(95% CI)** | **Adjusted model***  **OR (95% CI)** | **Adjusted final model****  **OR (95% CI)** |
| Maternal age (years) |  |  |  |
| <25 | 0.57 (0.18-1.78) | 0.98 (0.26-3.68) | NI |
| 25-35 | Ref | Ref | NI |
| >35 | 2.26 (072-7.02) | 2.19 (0.59-8.13) | NI |
| Mother-tongue |  |  |  |
| Norwegian | Ref | Ref | NI |
| Non-Norwegian | 3.28 (0.72-14.99) | 2.19 (0.49-9.79) | NI |
| Education |  |  |  |
| School/High school | 0.60 (0.29-1.21) | 0.80 (0.36-1.79) | NI |
| College/University | Ref | Ref | NI |
| Marital status |  |  |  |
| Married/co-inhabiting | Ref | NI | NI |
| Single/separated/divorced/other | 0.68 (0.19-2.46) | NI | NI |
| Occupation |  |  |  |
| Student/homemaker | 0.35 (0.12-1.02) | 0.46 (0.13-1.66) | NI |
| Healthcare professional | 0.83 (0.33-2.11) | 0.88 (0.31-2.49) | NI |
| Other paid work | Ref | Ref | NI |
| Unemployed/Sick leave/Social support | 0.43 (0.17-1.09) | 0.61 (0.23-1.61) | NI |
| Planned pregnancy |  |  |  |
| Yes | Ref | NI | NI |
| No | 1.81 (0.37-8.71) | NI | NI |
| No, but it was not unexpected | 0.56 (0.24-1.28) | NI | NI |
| BMI |  |  |  |
| Underweight | 1.69 (0.18-15.9) | NI | NI |
| Normal | Ref | NI | NI |
| Overweight | 1.08 (0.55-2.12) | NI | NI |
| Parity |  | NI | NI |
| Nulliparous | Ref | NI | NI |
| Multiparous | 0.93 (0.47-1.87) | NI | NI |
| Smoking in pregnancy |  | NI | NI |
| Yes | 0.99 (0.23-4.23) | NI | NI |
| No | Ref | NI | NI |
| Preferable psychiatric treatment |  |  |  |
| Treatment with AD/Combination of psychotherapy and pharmacological treatment | Ref | Ref | Ref |
| Non-pharmacological treatment/ No treatment/ Unsure | 2.38 (1.19-4.74) | 2.00 (0.84-4.72) | 2.26 (1.13 – 4.49) |
| Psychotherapy |  |  |  |
| No previous or current psychotherapy | 1.77 (0.90-3.51) | 1.57 (0.76-3.23) | NI |
| Received before, during or after pregnancy | Ref | Ref | NI |
| AD use |  |  |  |
| Non-users or discontinuers before pregnancy | 1.10 (0.56-2.16) | NI | NI |
| Continuers or initiators in pregnancy, or re-initiators postpartum | Ref | NI | NI |
| Perception of benefit-risk differential of AD in pregnancy | 0.95 (0.88-1.02) | NI | NI |
| Number of mental illnesses | 0.92 (0.66-1.30) | NI | NI |
| Depressive symptoms |  | NI | NI |
| PHQ-2 (≥1) Yes vs No | 0.65 (0.32-1.31) | NI | NI |
| EDS (≥13), Yes vs No | 1.24 (0.61-2.52) | NI | NI |
| Doctor-patient relationship | 1.22 (1.01-1.46) | 1.17 (0.95-1.43) | 1.20 (1.00 – 1.44) |
| Partner support | 1.45 (0.81-2.59) | 1.11 (0.60-2.07) | NI |

NI = not included. *Indicates the multivariable model where all maternal factors having a p-value < 0.2 in the univariate analysis (shown as crude odds ratio) were included. **Indicates the final multivariable model where only predicting variables were retained.

**Supplementary 7**: Results of the univariate and the full multivariable logistic regression for the association between moderate to high DCS and maternal factors in the postnatal sample

| Maternal factor |  | | |
| --- | --- | --- | --- |
|  | **Crude OR**  **(95% CI)** | **Adjusted model***  **OR (95% CI)** | **Adjusted final model****  **OR (95% CI)** |
| Maternal age (years) |  |  |  |
| <25 | - | NI | NI |
| 25-35 | Ref | NI | NI |
| >35 | 0.57 (0.17-1.92) | NI | NI |
| Breastfeeding (current or previous) |  |  |  |
| Yes | Ref | Ref | NI |
| No | 0.75 (0.21-2.75) | NI | NI |
| Education |  |  |  |
| School/High school | 1.50 (0.56-4.04) | NI | NI |
| College/University | Ref | NI | NI |
| Marital status |  |  |  |
| Married/co-inhabiting | Ref |  | NI |
| Single/separated/divorced/other | 1.18 (0.12-11.97) |  | NI |
| Occupation |  |  |  |
| Student/homemaker | 1.83 (0.36-9.41) | 1.67 (0.28-9.99) | NI |
| Healthcare professional | 0.48 (0.15-1.51) | 0.54 (0.17-1.72) | NI |
| Other paid work | Ref | Ref | NI |
| Unemployed/Sick leave/Social support | 0.39 (0.12-1.28) | 0.44 (0.09-2.01) | NI |
| Planned pregnancy |  |  |  |
| Yes | Ref | NI | NI |
| No | 2.63 (0.53-12.9) | NI | NI |
| No, but it was not unexpected | 1.79 (0.53-6.07) | NI | NI |
| BMI |  |  |  |
| Underweight | - | - | NI |
| Normal | Ref | NI | NI |
| Overweight | 1.16 (0.48-2.81) | NI | NI |
| Parity |  | NI | NI |
| Nulliparous | Ref | NI | NI |
| Multiparous | 0.93 (0.47-1.87) | NI | NI |
| Smoking in pregnancy |  | NI | NI |
| Yes | 0.77 (0.07-9.01) | NI | NI |
| No | Ref | NI | NI |
| Preferable psychiatric treatment |  |  |  |
| Treatment with AD/Combination of psychotherapy and pharmacological treatment | Ref | Ref | Ref |
| Non-pharmacological treatment/ No treatment/ Unsure | 1.18 (0.46-3.04) | NI | NI |
| Psychotherapy |  |  |  |
| No previous or current psychotherapy | 0.68 (0.28-1.66) | NI | NI |
| Received before, during or after pregnancy | Ref | NI | NI |
| AD use |  |  |  |
| Non-users or discontinuers before pregnancy | 0.95 (0.38-2.37) | NI | NI |
| Continuers or initiators in pregnancy, or re-initiators postpartum | Ref | NI | NI |
| Perception of benefit-risk differential of AD in pregnancy | 1.00 (0.93-1.08) | NI | NI |
| Number of mental illnesses |  |  |  |
| Only 1 | Ref | Ref | NI |
| >1 | 2.84 (1.04-7.75) | 2.69 (0.95-7.59) | NI |
| Depressive symptoms |  | NI | NI |
| PHQ-2 (≥1) Yes vs No | 1.38 (0.57-3.32) | NI | NI |
| EDS (≥13), Yes vs No | 1.52 (0.62-3.76) | NI | NI |
| Doctor-patient relationship | 1.40 (1.08-1.82) | 1.42 (1.06-1.91) | 1.40 (1.08-1.82) |
| Partner support | 0.96 (0.61-1.52) | NI | NI |

NI = not included. *Indicates the multivariable model where all maternal factors having a p-value < 0.2 in the univariate analysis (shown as crude odds ratio) were included. **Indicates the final multivariable model where only predicting variables were retained.
